# Risk Factors of Synchronous Inguinal Lymph Nodes Metastasis for Lower Rectal Cancer Involving the Anal Canal

**DOI:** 10.1371/journal.pone.0111770

**Published:** 2014-11-19

**Authors:** Renjie Wang, Peng Wu, Debing Shi, Hongtu Zheng, Liyong Huang, Weilie Gu, Ye Xu, Sanjun Cai, Guoxiang Cai

**Affiliations:** 1 Department of Colorectal Surgery, Fudan University Shanghai Cancer Center, Shanghai, China; 2 Department of Oncology, Shanghai Medical College, Fudan University, Shanghai, China; University General Hospital of Heraklion and Laboratory of Tumor Cell Biology, School of Medicine, University of Crete, Greece

## Abstract

**Purpose:**

The aim of the study is to identify the risk factors of synchronous ILN metastasis for lower rectal cancer involving the anal canal.

**Methods:**

Patients with lower rectal cancer who underwent radical resection at the Fudan University Shanghai Cancer Center were retrospectively analyzed. The synchronous ILN metastasis was defined as the metastasis occurring within 6 months after the diagnosis of rectal cancer. Patients’ gender, age, tumor diameter, dentate line invasion, differentiation level, histological type, depth of invasion, perirectal LN metastasis, lymphovascular invasion or perineural invasion were analyzed in the study. The correlation between synchronous ILN involvement and clinicopathological features were analyzed with Chi-square test/fisher’s exact test. Variables with p<0.05 in univariate analysis were then analyzed in a multivariate logistic model. Odds ratio (OR) along with 95% confidence intervals (95% CI) were calculated.

**Results:**

A total of 325 patients (182 men and 143 women) with lower rectal cancer met the criteria and were enrolled in the study. Among them, 20 patients (6.2%) had synchronous ILN metastasis. Both univariate and multivariate analysis showed the invasion of the dentate line had a strong correlation with synchronous ILN metastasis with the odds ratio (OR) of 23.558 [95% confidence interval (CI) 6.380–86.982] (p<0.001). The presence of lymphovascular invasion also showed a significant correlation synchronous ILN metastasis with odds ratio (OR) of 5.260 [95% confidence interval (CI) 1.818–15.212] (p = 0.002).

**Conclusions:**

The invasion of dentate line and lymphovascular invasion are two independent risk factors of inguinal lymph node metastasis for lower rectal cancer involving the anal canal.

## Introduction

Inguinal lymph nodes (ILN) metastases from rectal cancer are relatively rare [1], [2]. They are believed to arise from advanced primary lesions with proximal lymphatic obstruction which results retrograde nodal spread, or from recurrent disease in the pelvis or perineum [3]. In a retrospective study of 32 patients with ILN metastases from rectal cancer, 0% 5-year overall survival was observed, which showed poor prognosis of ILN metastases in rectal cancer. Lymphatic spread from tumors located in the lower rectum are also complex and unpredictable [2]. Therefore, the prevention for ILN metastases is extremely critical for patients with lower rectal cancer. Compared to penile cancer and perineal cancer, fewer studies were related with ILN metastasis in lower rectal cancer. In this study, we performed a retrospective study of a cohort of lower rectal cancer patients and analyzed the potential predictors of synchronous ILN metastasis.

## Materials and Methods

Patients with lower rectal cancer who underwent abdominal perineal resection (APR) at the Fudan University Shanghai Cancer Center from September 1986 to July 2013 were reviewed in this study. None of the patients received any preoperativechemoradiotherapy at operation. Written informed consent was obtained from all study participants adhering to the local ethical guidelines prior to specimen collection. The study protocol and consent procedure were approved the Ethics Committee of the Shanghai Cancer Center, Fudan University.

All the patients met the criteria as follows: (1) the lower edge of the tumor located less than 2 cm proximal to the dentate line on digital rectal examination and then was confirmed by postoperative pathological record; (2) having complete medical records including demographic information, clinical and pathological data, operation notes, and follow-up results; and (3) having at least 6 months follow-up time after the operation.

Patients who (1) had ILN metastasis occurring 6 months after the diagnosis of rectal cancer (61 patients); (2) had incomplete medical records (18 patients); (3) had other histological type including squamous carcinoma, carcinoid and melanoma (5 patients) were all excluded from the study. ILN Metastases were suspected either by palpable lymph nodes or by image results before or after the surgical procedure. The synchronous ILN metastasis was defined as the metastasis occurring within 6 months after the diagnosis of anorectal cancer and confirmed by pathology with the inguinal lymph nodes resection or biopsy or fine needle aspiration. The cancer staging was based on the American Joint Committee on Cancer 7th edition.

Patient’s gender, age, tumor diameter, dentate line invasion, differentiation level, histological type, depth of invasion, perirectal LN metastasis, lymphovascular invasion or perineural invasion were analyzed in the study. ILN metastasis was not defined as perirectal LN metastasis.

The correlation between synchronous ILN involvement and clinicopathological features were analyzed with Chi-square test/fisher’s exact test. Variables with p<0.05 in univariate analysis were then analyzed in a multivariate logistic model (using binary logistic, step backward method). Values of p<0.05 was considered statistically significant, and odds ratio (OR) along with 95% confidence intervals (95% CI) were calculated. All statistical analysis were performed by SPSS, version 19.0.0 (SPSS, Inc., Chicago, IL, USA).

## Results

### General information of patients

A total of 325 patients (182 men and 143 women) with lower rectal cancer met the criteria and were enrolled in the study. The median age at the time the diagnosis of rectal cancer was 56 years (range, 25–86 years). The median maximum diameter of the tumor was 36.0 mm (range, 8–100 mm). Among them, 20 patients (6.2%) were confirmed with synchronous ILN metastasis (Table 1). The clinical and pathological data and postoperative treatment information of the 20 patients with ILN metastasis is shown in Table 2.

**Table 1:** General information of patients (n = 325).

Characteristic	Cases (%) (n = 325)
Gender	
Male	182 (56.0%)
Female	143 (44.0%)
Median age (years) at diagnosis (range)	56.0 (25–86)
Dentate line invasion	
Yes	89 (27.4%)
No	236 (72.6%)
Maximum diameter (mm) of tumor (range)	36.0 (8–100)
Histological type	
Adenocarcinoma	264 (81.2%)
Mucinous	57 (17.6%)
Signet-cell	4 (1.2%)
Differentiation	
Poor	107 (32.9%)
Moderate	215 (66.2%)
Well	3 (0.9%)
Depth of invasion	
T1	9 (2.8%)
T2	122 (37.5%)
T3	56 (17.2%)
T4	138 (42.5%)
Perirectal LNM	
Yes	146 (44.9%)
No	179 (55.1%)
Lymphovascular invasion	
Yes	70 (21.5%)
No	255 (78.5%)
Perineural invasion	
Yes	78 (24.0%)
No	247 (76.0%)
Synchronous ILN metastasis	
Yes	20 (6.2%)
No	305 (93.8%)

**Table 2:** Clinicopathological profile and postoperative treatments of the 20 patients with synchronous ILN metastasis.

No.	Gender	Age(year)	Maximumdiameter(mm)	Dentatelineinvasion	Histologicaltype	Differentiation	Depth ofinvasion	PerirectalLNmetastasis(+/−)	LVI(+/−)	Perineuralinvasion	Unilateral/BilateralILN metastasis	Treatments
1	Female	77	12	Yes	Mucinous	Poor	T2	+	+	+	Right side	R→D
2	Female	48	30	Yes	Mucinous	Poor	T2	+	+	−	Left side	PC
3	Male	63	40	Yes	Adenocarcinoma	Moderate	T4	−	−	−	Left side	D
4	Male	57	37	No	Adenocarcinoma	Poor	T4	−	+	−	Left side	D
5	Male	65	20	Yes	Mucinous	Poor	T3	+	+	+	Right side	D
6	Male	62	20	Yes	Adenocarcinoma	Moderate	T4	−	+	−	Right side	D
7	Female	34	40	No	Adenocarcinoma	Poor	T4	+	+	−	Right side	R
8	Female	46	12	Yes	Adenocarcinoma	Moderate	T4	−	−	−	Right side	D
9	Male	51	45	No	Mucinous	Poor	T3	+	−	−	Bilateral	D
10	Male	70	35	Yes	Signet	Poor	T4	−	−	−	Left side	D
11	Male	33	40	Yes	Adenocarcinoma	Poor	T2	−	−	−	Left side	D
12	Male	56	20	Yes	Adenocarcinoma	Moderate	T4	−	−	+	Right side	D
13	Male	60	30	Yes	Adenocarcinoma	Moderate	T3	−	−	−	Right side	R
14	Female	59	35	Yes	Adenocarcinoma	Moderate	T4	−	−	+	Left side	R
15	Male	62	45	Yes	Adenocarcinoma	Moderate	T3	+	+	−	Left side	D
16	Male	61	50	Yes	Adenocarcinoma	Poor	T3	−	−	−	Right side	PC
17	Male	39	50	Yes	Mucinous	Poor	T2	−	−	−	Bilateral	PC
18	Male	42	35	Yes	Adenocarcinoma	Poor	T3	+	+	+	Left side	PC
19	Male	70	25	Yes	Adenocarcinoma	Moderate	T3	−	−	−	Right side	D
20	Female	37	26	Yes	Adenocarcinoma	Moderate	T4	−	+	+	Left side	D

LVI = Lymphovascular Invasion, R = Radiation Therapy, D = Dissection, C = Palliative Chemotherapy.

### Univariate analysis of risk factors for lower rectal cancer involving the anal canal

The associations between synchronous ILN metastasis and clinicopathological features are shown in Table 3. The univariate analysis showed factors including dentate line invasion, perirectal LN metastasis and lymphovascular invasion were significantly related to the synchronous ILN metastasis. No significant correlation was seen between synchronous ILN metastasis and patients’ gender, age, tumor diameter, histological type, T stage or presence of perineural invasion.

**Table 3:** Association between clinicopathological factors and synchronous ILN metastasis.

Characteristic	Synchronous ILN Metastasis		P Value
	Yes (n = 20)	No (n = 305)	
Gender			0.247
Male	14 (70.0%)	168 (55.1%)	
Female	6 (30.0%)	137 (44.9%)	
Age			0.635
≥60 years	9 (45.0%)	114 (37.4%)	
<60 years	11 (55.0%)	191 (62.6%)	
Dentate line invasion			**<0.001**
Yes	17 (85.0%)	72 (23.6%)	
No	3 (15.0%)	233 (76.4%)	
Maximum diameter			0.816
≥30 mm	11 (55.0%)	180 (59.0%)	
<30 mm	9 (45.0%)	125 (41.0%)	
Histological type			0.140
Adenocarcinoma	14 (70.0%)	250 (82.0%)	
Mucinous	5 (25%)	52 (17%)	
Signet	1 (5%)	3 (1%)	
Differentiation			0.102
Poor	11 (55.0%)	96 (31.5%)	
Moderate	9 (45.0%)	206 (67.5%)	
Well	0 (0%)	3 (1%)	
T stage			0.062
T1–2	4 (20.0%)	127 (41.6%)	
T3–4	16 (80.0%)	178 (58.4%)	
Perirectal LNM			**0.022**
Positive	14 (70.0%)	132 (43.3%)	
Negative	6 (30.0%)	173 (56.7%)	
LVI			**0.019**
Yes	9 (45.0%)	61 (20.0%)	
No	11 (55.0%)	244 (80.0%)	
Perineural invasion			
Yes	6 (30.0%)	72 (23.6%)	0.705
No	14 (70.0%)	233 (76.4%)	

### Multivariate analysis of risk factors for lower rectal cancer involving the anal canal

Variables with p<0.05 in univariate analysis were then analyzed. Multivariate logistic analysis was performed (Table 4) with the factor including dentate line invasion, perirectal LN metastasis and lymphovascular invasion. The invasion of the dentate line showed a strong correlation with synchronous ILN metastasis with the odds ratio (OR) of 23.558 [95% confidence interval (CI) 6.380–86.982] (p<0.001). The presence of lymphovascular invasion also showed a significant correlation synchronous ILN metastasis with odds ratio (OR) of 5.260 [95% confidence interval (CI) 1.818–15.212] (p = 0.002). The results indicated invasion of the dentate line and lymphovascular invasion are two independent risk factors for lower rectal cancer involving the anal canal.

**Table 4:** Multivariate analysis.

Characteristic	n	OR	95% CI	P
Dentate line invasion				
No (Referent)	236	1.0		
Yes	89	22.759	6.137–84.404	<0.001
Lymphovascular invasion				
No (Referent)	255	1.0		
Yes	70	4.157	1.317–13.122	0.015
Perirectal LNM				
No (Referent)	179	1.0		
Yes	146	1.753	0.562–5.467	0.034

## Discussion

The rectum is divided into upper and lower segments according to the relative location from the peritoneal reflection by convention. In Japan, the lower rectum is further divided into two subregions, i.e. ‘Rb tumors’ and ‘Rp tumors.’ An ‘Rb tumor’ indicates a tumor located in the lower rectum without any invasion of the dentate line. An ’Rp tumor’ indicates a tumor invades the dentate line [4]. In a Japanese study of 156 lower rectal cancer patients, 28% (7/25) of ‘Rp tumor’ patients had ILN metastasis. ‘Rp tumor’ was found associated with high rate of ILN metastasis, and also had worse prognosis and local recurrence than ‘Rb tumor’. And the presence of poorly differentiated or mucinous adenocarcinoma is a risk factor of local recurrence for 25 ‘Rp tumor’ patients [5]. In our studies, there are 83 patientsmet the criteria of ‘Rp tumor.’ And our results showed ‘Rp tumor’, invading the dentate line, is an independent risk factor for lower rectal cancer involving the anal canal. This can be explained that in a tumour that originates above the dentate line the lymphatics drain mostly to the mesenteric lymph nodes (MLN) and lateral lymph nodes (LLN), while below the dentate line the lymphatics drain mostly to the inguinal lymph nodes (ILN) [6]. Moreover, in our study, the presence of lymphovascular invasion of lower rectal cancer is shown to be another independent risk factor. The result is consistent with the lymphatic anatomy. Also, we found no significant correlation between synchronous ILN metastasis and patients’ gender, age, tumor size, histological type, T stage or presence of perineural invasion, which was never revealed in other studies.

For all patients underwent digital rectal examination, their tumor location will be again confirmed by postoperative pathological record. As for the tumors which overlap the anorectal junction, the determination of the anatomy can be ambiguous. In our study, we used the classification from AJCC. According to AJCC, if epicenter of a tumor is located more than 2 cm proximal to the dentate line or proximal to the anorectal ring on digital rectal examination, such tumor should be classified as rectal cancer [7].

Compared to rectal cancer, the ILN metastasis is more common in anal canal carcinoma with 5–25% patients [8]–[12]. In a cohort study of 206 lower rectal cancer patients, Bebenek Marek et al reported ILN metastasis with only 2.9% (6/206) patients [13].

Although there are few reports about ILN metastasis for rectal cancer, the outcome of poor prognosis of ILN metastasis for rectal cancer is clear [13], [14]. Therefore, prevention or early diagnosis of ILN metastasis for lower rectal cancer is very critical. Although PET-CT scan is useful for staging and finding metastatic lesions, according to NCCN guidelines version 3.2014 for rectal cancer, it does not supplant a contrast-enhanced diagnositic CT scan. PET-CT should only be used to evaluate an equivocal finding on a contrast-enhanced CT scan or in patients with strong contraindications to IV contrast. And for the equivocal findings on a contrast-enhanced CT scan, aspiration or biopsy is still the gold standard, the specificity and positive predictive value of PET-CT is only 83% and 43% respectively [15]. In our institution, ILN dissection and postoperative ILN irradiation is the conventional treatment for synchronous ILN metastasis for lower rectal cancer. In patients confirmed with synchronous ILN metastasis, 12 patients (60%) received ILN dissection, 3 patients (15%) received postoperative ILN irradiation, 1 patient (5%) received both irradiation and ressection and 4 patients (20%) were given palliative chemotherapy because of systemic metastases (Table 2). The role of preventive treatment for ILN metastasis for lower rectal cancer has been not well defined yet. The complication of ILN dissection includes lymphoedema and lymphocele [16]–[18] and prophylactic ILN dissection is also not a routine treatment for lower rectal cancer. In our study, the dentate line invasion and lymphovascular invasion were shown to be critical risk factors for synchronous ILN metastasis for lower rectal cancer. Therefore, for those patients with those risk factors, prophylactic ILN dissection could be considered to prevent the ILN metastasis. As for the radiotherapy, there is little information about the preventive irradiation either. A study from M.D. Anderson claimed that inguinal nodal failure in rectal cancer patients with anal canal involvement treated with neoadjuvant or adjuvant chemoradiation is not high enough to justify routine elective groin irradiation [19]. In 2010 clinical practice guideline for rectal cancer, experts from ESMO believed medial inguinal nodes need only be included prophylactically for radiotherapy when the tumor grows at or below the dentate line [20].

Moreover, we had to admit there was still limitation in our study. The sample group is small with only 20 patients included. Therefore, a larger size of sample with multiple centers is called for further study. And patients with metachronous ILN metastasis (ILN metastasis occurring over 6 months after the diagnosis of rectal cancer) will be analyzed in the following study, which would give alternative approach in prophylactic treatment of ILN metastasis for lower rectal cancer involving the anal canal.

## Conclusion

The invasion of dentate line and lymphovascular invasion are two independent risk factors of inguinal lymph node metastasis for lower rectal cancer involving the anal canal. For patients with these risk factors, radiological assessment for inguinal region and biopsy for inguinal lymphadenopathy are recommended.
